# Immunophenotypes in psychosis: is it a premature inflamm-aging disorder?

**DOI:** 10.1038/s41380-024-02539-z

**Published:** 2024-03-26

**Authors:** Song Chen, Yunlong Tan, Li Tian

**Affiliations:** 1 Peking University HuiLongGuan Clinical Medical School, Beijing Huilongguan Hospital, Beijing, PR China; 2 Department of Psychology and Logopedics, Faculty of Medicine, University of Helsinki, Helsinki, Finland

**Keywords:** Neuroscience, Schizophrenia

## Abstract

Immunopsychiatric field has rapidly accumulated evidence demonstrating the involvement of both innate and adaptive immune components in psychotic disorders such as schizophrenia. Nevertheless, researchers are facing dilemmas of discrepant findings of immunophenotypes both outside and inside the brains of psychotic patients, as discovered by recent meta-analyses. These discrepancies make interpretations and interrogations on their roles in psychosis remain vague and even controversial, regarding whether certain immune cells are more activated or less so, and whether they are causal or consequential, or beneficial or harmful for psychosis. Addressing these issues for psychosis is not at all trivial, as immune cells either outside or inside the brain are an enormously heterogeneous and plastic cell population, falling into a vast range of lineages and subgroups, and functioning differently and malleably in context-dependent manners. This review aims to overview the currently known immunophenotypes of patients with psychosis, and provocatively suggest the premature immune “burnout” or inflamm-aging initiated since organ development as a potential primary mechanism behind these immunophenotypes and the pathogenesis of psychotic disorders.

## Introduction

Immune processes have long been considered a potential initiating environmental insult in psychiatric disorders including schizophrenia. The past three decades have witnessed rapidly accumulating evidence on the aberrant roles that various immune cells may play in psychiatric disorders, covering both lymphocyte-oriented adaptive immune hypothesis and microglia/macrophage-oriented innate immune hypothesis as well as hypotheses on imbalanced ratios of innate or adaptive immune subsets. As such, the potential clinical implications of immune components have attracted intensive interest in the research field of “immunopsychiatry” [1].

Despite this advancement, some vital questions, such as whether immune cells are more activated or vice versa in psychosis, whether the observed immune changes are the reason or sequelae of psychosis, which immune pathways or biomarkers are specific for a certain type of psychiatric disorder, and whether peripheral inflammation reflects inflammatory status of the brain, have remained vague or even controversial. White blood cells (WBCs, e.g., blood leukocytes) are a group of enormously plastic and heterogeneous immune cells, encompassing a vast range of lineages and sub-lineages phenotypically and functionally, rendering a full comprehension of their diverse roles in psychiatric disorders a challenging task. This review aims to holistically summarize the currently known immunophenotypes of circulatory WBCs and their brain counterparts in psychosis, highlighting mismatches among these immunophenotypes, and propose inflamm-aging as a potential underlying mechanism for the heterogeneous mismatches between the peripheral innate and adaptive immune components, as well as those between the blood and the brain.

The term “Inflammation-ageing” emerged in the early 2000s to indicate a widespread immune dysregulation in the elderly, which is represented by persistently increased proinflammatory mediators and yet accompanied with immunosenescence or reduced immune responsiveness to immunogenic triggers [2]. Inflamm-aging is confirmed as a risk factor for many chronic degenerative diseases including cardiovascular disease, metabolic syndrome, cancer, and dementia [3–5]. Also, autoantibody-mediated cognitive impairment, which is regarded as a precursor to dementia and delirium, could also be caused by inflamm-aging [6–9]. Coincidently, people with major psychiatric disorders have averagely 15~20-year shorter life span than the general population, mainly due to comorbid cardiovascular and metabolic diseases [10]. This suggests that psychosis and metabolic disturbances may share certain genetic or pathobiological risks, such as inflammatory mechanisms [11, 12]. Yet, we think that inflamm-aging should not be merely regarded as a collateral result of these comorbid diseases in psychotic disorders. On the contrary, inflamm-aging may represent a primary immune mechanism that contributes to pathophysiology of psychosis and associated cognitive deficits, which has not been paid attention to in this research field. More importantly, we disseminate immunophenotypes of psychosis and their link to inflamm-aging from a potential perspective of developmental roots of inflamm-aging in psychosis. To comply with space limit and create a relatively simple scenario, we would like to exclude psychotic conditions with affective components such as bipolar disorder and focus on psychosis, especially schizophrenia, at first-episode/acute/early stage for our arguments.

## WBCs in psychosis

**Fig. 1:**
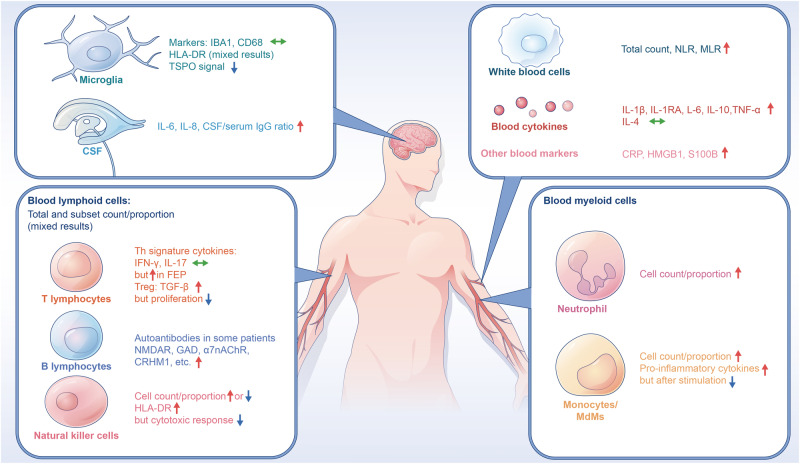
Immunophenotypes in psychosis. There are two facets of mismatched systemic changes of immune cells in patients of psychosis. On one facet, blood cytokines and numbers of leukocytes and their innate subsets such as neutrophils and monocytes are increased while activation of innate cells including monocytes and innate lymphoid cells such as NK cells after stimulation are decreased. On the other facet, the mononuclear phagocytic system inside and outside the brain, such as microglia and monocytes, are not synchronically activated in psychosis. That is, there is no overt activation of myeloid cells in the psychotic brain as compared to the immune changes observed in the blood and CSF. α7nAChR α7 nicotinic acetylcholine receptor, CD cluster of differentiation, CRHM1 cholinergic receptor muscarinic 1, CRP C-reactive protein, CSF cerebrospinal fluid, FEP first-episode psychosis, GAD glutamic acid decarboxylase, HLA human leukocyte antigen, HMGB1 high mobility group box 1, IBA1 ionized calcium-binding adapter molecule 1, IFN interferon, IL interleukin, MLR monocyte to lymphocyte ratio, NK natural killer, NLR neutrophil to lymphocyte ratio, NMDAR N-methyl-D-aspartate receptor, S100B S100 calcium-binding protein B, TGF transforming growth factor, TNF tumor necrosis factor, TSPO translocator protein. Green arrows represent no change, blue arrows represent downregulation, and red arrows indicate upregulation.

Multiple studies have consistently reported increases in total and/or differential WBC counts including neutrophils and monocytes, as well as alterations in cytokine production in patients with psychosis, including first-episode psychosis (FEP) or first-episode schizophrenia (FES), compared to healthy controls (HCs) [13–17] (Fig. 1, Tables 1 & S1). The most recent meta-analyses showed that compared to HCs, total WBC count was increased in psychosis patients and so was monocytic count (i.e., monocytosis) in FEP/FES patients with small-medium effect sizes [14, 18]. Some studies have also examined the cerebrospinal fluid in psychotic patients and found accumulation of monocytes/macrophages in patients during acute psychotic episode or with positive psychotic symptoms [19, 20]. Associations of WBC count and monocytosis with worsening of psychotic symptoms have been found as well, including FEP [21].

**Table 1:** Overview of immunophenotypes in first-episode or acute psychosis.

Literature type	Study indicators	Results			Associations with clinical indices	Consistency to inflamm-aging
		Significant changes	Increased	Decreased		
**Observational study:** drug-naïve FEP patients (*n* = 129); non-first-episode SCZ patients unmedicated ≥6 weeks (*n* = 124); HC (*n* = 294) [21]	Differential blood count, CRP, neutrophil and monocyte–macrophage activation markers, cortisol, and psychotic symptoms in acute FEP and SCZ	Neutrophils, monocytes, and CRP (acutely ill unmedicated FEP vs. HC)	√		- Positive correlations of neutrophils, monocytes, and CRP with PANSS positive symptoms in FEP; - Improvement of positive symptoms after treatment correlated with decline of neutrophils or CRP and rise of eosinophils	Yes [3–5]
		Eosinophils (acutely ill unmedicated FEP vs. HC)		√		? [180]
**Observational study:** FEP (*n* = 137); HC (*n* = 81) [24]	Blood cells, gray matter, and ventricles in FEP	Neutrophils (FEP vs. HC)	√		- Neutrophil count was associated with reduced gray matter volume and increased cerebrospinal fluid volume; - Positive association of neutrophil count with PANSS total score	Yes [178]
**Observational study:** SCZ (*n* = 9, young drug-free men with SCZ); HC (*n* = 11) [36]	Ultrastructure of monocytes and monocytic production of IL-1β	Area of nucleolus, volume density and area of mitochondria and lysosomes, and the number of lysosomes (SCZ vs. HC)	√		None of the parameters were correlated with PANSS scores.	Yes [181]
		Production of IL-1β by unstimulated monocytes (SCZ vs. HC)	√			Yes [181]
**Observational study:** FES (*n* = 69, patients with normal weight, drug naïve); HC (*n* = 60) [73]	Proportion of Th-17 cells and plasma levels of IL-17, IFN-γ, and IL-6	Proportion of Th-17 cells (FES vs. HC)	√		- Positive correlations of Th-17 cells, IL-17, IFN-γ, and IL-6 with PANSS total score	Yes [182]
		Plasma levels of IFN-γ and IL-6 (FES vs. HC)	√			Yes [5]
**Observational study:** FEP (*n* = 81); HC (*n* = 61) [83]	NK cell phenotype and function	NK cell expression of HLA-DR (BP and FEP vs. HC)	√		- Positive correlation of NKG2C with YMRS score in FEP; - Inverse correlation of NK cell, IFN-γ, with PANSS scores in FEP	Yes? [183]
		Expression of the activating NKG2C receptor (FEP vs. HC)	√			Yes? [184]
		Capacity of NK cells to mount cytotoxic responses (FEP vs. HC)		√		Yes [183, 184]
**Meta-analysis** (10 studies) [25]	Twenty‐four peripheral cytokines	IFN‐γ, IL‐6, IL‐12 and IL‐17 (Antipsychotic-naïve FEP vs. HC)	√		- Positive correlation of IL‐1β, IL‐2, IL‐6, and TNF‐α with PANSS negative symptoms; - Negative and positive correlation of IL‐10 and IL-4 with PANSS negative symptoms, respectively	Yes (IFN‐γ, IL‐6, and IL-17) [5, 185]; Yes? (IL-12) [181, 186]
**Observational study:** FES (*n* = 110); HC (*n* = 50) [79]	Serum anti-NMDA receptor antibody	Serum anti-NMDAR antibody (FES vs. HC)	√		- Positive correlation with PANSS total and subdomain scores; - Negative correlation with MCCB total and subdomain scores	Yes [187]

Inflammatory indices, their associations with psychotic symptoms, and the consistency of their changes to inflamm-aging phenomenon are summarized (see also Table S1).

*BP* bipolar disorder, *CRP* C-reactive protein, *FEP* first-episode psychosis, *FES* first-episode schizophrenia, *HC* healthy control, *HLA* human leukocyte antigen, *IFN* interferon, *IL* interleukin, *MCCB* MATRICS Consensus Cognitive Battery, *NK* natural killers, *NMDAR* N-methyl-D-aspartate receptor, *PANSS* positive and negative symptoms scale, *SCZ* schizophrenia, *Th* T helper cells, *TNF* tumor necrosis factor, *YMRS* Young mania rating scale.

Besides, relative neutrophil to lymphocyte ratio (NLR) and monocyte to lymphocyte ratio (MLR) are elevated in FEP/FES as well [22, 23] and have been reported to be positively correlated with cytokines and acute phase reactants such as C-reactive protein (CRP), which are also altered in FEP/FES, and with cortical gray matter dystrophy and/or severity of psychotic symptoms in psychotic patients, including FEP [21, 24, 25]. Moreover, as indicators of endothelial dysfunction in cardiovascular and metabolic diseases, these leukocyte ratios may be useful to estimate long-term clinical outcomes in FEP/FES [13, 15].

Genome-wise association studies (GWAS) and epigenome-wide association studies (EWAS) of peripheral WBCs have also disseminated immune architecture of psychosis. As such, evidence from GWAS has substantiated the involvement of immune variants such as *HLA* and *C4* genes and immune pathways such as transforming growth factor (TGF)-β signaling and B-cell activation in schizophrenia [26]. Recently, genetic loci for the WBC count and WBC subtype counts were found to be shared with schizophrenia, particularly for lymphocytes [27]. Another GWAS unraveled causal contribution of lymphocytes to psychiatric disorders and genetic pathways shared by schizophrenia with autoimmune diseases and coronary heart disease [28], both known comorbidities of schizophrenia [10, 29]. Genetic single-nucleotide polymorphisms (SNPs) of multiple other cytokines may also be causally associated with schizophrenia [30]. Furthermore, genetic variants underlying the cortical size were significantly enriched in biological pathways of inflammation in subset of schizophrenia patients [31].

EWAS studies have also highlighted WBCs in psychosis. For instances, changes of DNA methylation in genes involved in T-cell development in schizophrenia patients was reported [32]. A cell-type-specific EWAS study of neonatal blood from individuals who developed schizophrenia later in life strongly supported the involvement of aberrant methylation in B-cells and the gene *BDNF* in schizophrenia susceptibility [33]. Besides, a most recent meta-analysis of the newborn cord blood observed the association of cumulative maternal stress with differential CpG methylation of genes implicated in immune cellular functions and schizophrenia risk, etc. [34]. By focusing on FES patients, we recently also showed that aberrant peripheral blood DNA methylations of neurodevelopmental genes were possibly involved in pathogenesis of schizophrenia [35].

## Monocytes and monocyte-derived macrophages (MdMs) in psychosis

### Circulatory monocytes/MdMs in psychosis

Studies have revealed cellular and molecular changes indicative of functional alterations of monocytes or MdMs in psychotic patients (Fig. 1). For instance, Uranova et al. found ultrastructural abnormalities of blood monocytes in a group of young drug-free schizophrenia patients by electron microscopy, including significantly increased volume densities and/or sizes of the subcellular organelles including lysosomes, mitochondria, and nucleolus; furthermore, production of the cytokine interleukin (IL)-1β by monocytes isolated from PBMCs was higher in patients than in HCs and was positively correlated with the volume and area of lysosomes indicating overactivation of monocytes [36]. Corroboratively, overexpression of this and other proinflammatory cytokines including IL-6 and tumor necrosis factor (TNF) in isolated CD14^+^ (classical) monocytes from schizophrenia patients was also found by earlier studies, showing particularly evident in patients with active psychosis [37]. In another recent study, elevated level of soluble CD14 in blood samples drawn from individuals who were subsequently diagnosed with schizophrenia was detected, implicating an advantageous monocytic activation before disease onset [38].

Müller et al. instead observed dampened cytokine production in CD14^+^ monocytes of schizophrenia patients after a *toll*-like receptor (TLR) ligand polyinosinic-polycytidylic acid (polyI:C) stimulation [39]. Likewise, a recent study reported that MdMs cultured from PBMCs of FEP patients had diminished inflammatory responses to lipopolysaccharide (LPS) plus IFNγ stimulation (inducing an M1 type) compared to HCs or affective FEP patients; furthermore, when skewed to an alternative (M2) type using LPS plus IL-4, MdMs from these patients also had dampened production of inflammatory cytokines compared to patients with affective FEP [40]. An earlier study also showed downregulated inflammatory genes in CD14^+^ monocytes isolated from schizophrenia patients compared to bipolar disorder [37]. Additionally, higher level of TLR-4 on CD14^+^ monocytes was detected in drug-naïve or chronic schizophrenia patients compared with HCs by two studies [39, 41] and was correlated with more severe cognitive deficits in drug-naïve schizophrenia patients [41], which however were not observed by us in FES patients [42]; interestingly, as compared to HCs, Müller et al. and we both observed more downregulated TLR-4 expression on CD14^+^ monocytes of schizophrenia patients after stimulation by polyI:C or LPS [39, 42], indicating blunted monocytic activation in patients.

One may wonder if the above-described studies had covered all monocytic subsets, which are known to play nonoverlapping functions. Monocytic subsets have not been meticulously studied in psychotic patients so far. Filling this gap in a small step, we recently observed that nonclassical monocytes were reduced in FES patients compared to HCs; furthermore, both classical and nonclassical monocytic signature genes were negatively associated with cerebral cortical thickness and cognitive performance in HCs, which was intriguingly weakened in FES patients [43].

### Brain monocytes/MdMs/microglia in psychosis

Unlike monocytes and MdMs (also referred as bone marrow-derived macrophages or microglia-like cells in the brain), most tissue-resident macrophages are established from the prenatal yolk sac and fetal liver and are self-renewed through adulthood. Notwithstanding this consensus, some findings cannot unequivocally exclude the possibility of at least partial monocytic origin of microglia during brain development [44, 45]. Among brain borderline- or barrier-associated macrophages (BaMs, located in the perivascular space, leptomeninges, and choroid plexus), BaMs in the choroid plexus are the only population with substantial constitution from bone marrow myeloid progenitors after birth [45, 46].

Although monocytes/MdMs do not penetrate the blood-brain barrier and contribute to the phagocytic cytoarchitecture of the healthy adult brain parenchyma, monocytes comprise ~30% of the cellular compartment in the normal adult cerebrospinal fluid [47], and interestingly a vast majority of infiltrated monocytes were found to be CD16^+^ (nonclassical) monocytes [48]. Furthermore, inflammatory MdMs can also infiltrate in the diseased brain parenchyma and phenocopy microglia, making their programming malleable to stimulus alike residential microglia. MdMs seem to be more efficient than microglia in particular tasks, e.g., migration toward inflammatory areas and phagocytic activities. However, whether MdMs can persist and become an integral part of the microglial pool after the resolution of brain inflammation and whether they can contribute to the molecular and functional heterogeneities of brain parenchymal macrophages is an ongoing debate still [44, 45].

Identifying similarities and differences among monocytes, MdMs, BaMs, and microglia is an important but not a trivial task, as myeloid signature markers are shared by these cells. Several transcriptomic studies nevertheless have shown that compared to circulating monocytes and macrophages, microglia have constitutively reduced expression of CD45 and were reported to express higher levels of CX3CR1, SIGLEC-H, FCRLS, and P2RY12 but lower levels of AIF1 (IBA1), CD11B, CD206, CSF1R, and F4/80 [44, 45]. It should be pointed out however that microglia from different brain regions express these genes at different levels under steady state, thereby complicating the ubiquitous uniqueness of these markers [49, 50].

Under psychotic conditions, discerning activated microglia from infiltrated MdMs can be even more challenging, as their inflammatory grade is usually low or even missing compared to neurological conditions [51, 52]. While it is still uncertain whether microglia are activated or not in the *post-mortem* brains of psychotic patients by immunohistochemistry [53] (Fig. 1), some studies found primed or reactive microglial subtypes in schizophrenia patients. For instance, HLA-DR^+^ microglia in the dorsolateral prefrontal cortex were found to be increased and reflect impaired cerebral lateralization in chronic schizophrenia [54]; likewise, a recent study on chronic schizophrenic patients with active psychosis found increased CD64^+^ microglia in the same brain region but no significant changes in BaMs [55]. Extending to studies on in vivo positron emission tomography (PET) imaging for the 18 kDa translocator protein (TSPO) in the living brains of psychotic patients, the field has generated mixed scenarios too, lacking solid evidence on myeloid activation and showing even consistently decreased rather than increased TSPO signal in psychosis including drug-free or recent-onset schizophrenia [56–59], which is also reminded (except in PET) in major depressive disorder [59, 60].

Evidence on differential molecular features of monocytes versus microglia in psychotic conditions is still sparce currently. A recent genetic study on human microglia identified a schizophrenia candidate gene *IFRD1* that is proposedly specific for microglia and may not be captured in monocytes [61]. Besides, the mood stabilizer lithium was previously reported to induce complement-3 production only in microglia and differentiated monocytic cells but not circulating monocytes [62].

Overall, evidence on circulatory and brain monocytic/microglial lineages indicates their complicated functions in psychosis. The discrepancies on cytokine production and activation state of blood monocytes/MdMs may be caused by different monocyte/MdMs subsets that play heterogeneous roles in the pathophysiological process of psychosis. Alternatively, these discrepancies may suggest inflamm-aging-like aberrant innate immune response or immunosuppression in primed monocytes/MdMs of psychotic patients [63], which we will further elaborate in “Potential mechanisms of immunophenotypes in psychosis: how to accommodate discrepancies?” section, when discussing on underlying mechanisms contributing to myeloid phenotypes both inside and outside the brain.

## Lymphocytes in psychosis

As active players in adaptive immunity and autoimmunity, blood lymphocytes have been suggested to contribute to the pathogenesis of schizophrenia, at least in subgroups of patients [29, 64] (Fig. 1). A recent study observed a population of CD69^+^ Th and regulatory T (Treg) cells in the brain parenchyma, which remained resident for some weeks in both mice and humans and helped microglia to complete the fetal-to-adult transition, the defect of which resulted in excess immature neuronal synapses as well as anxiety and cognitive impairment [65]. Another recent single-cell analysis revealed that T-cells can infiltrate into the subventricular zone from the lateral ventricles and inhibit proliferation of neural stem cells partly by secreting interferon (IFN)-γ in the aged mouse and human brains [66]. Perhaps such evidence can provide some new clue on the future imaging studies for psychosis.

While an old meta-analysis found significantly elevated total lymphocyte cell count and T-cell count in FEP [67], other studies found reduced T-cell numbers (i.e., lymphopenia) in acute paranoid schizophrenia (reviewed in [68]). A more recent meta-analysis did not find the total lymphocyte cell count significantly altered in FEP or drug-naïve schizophrenia patients but noted elevated blood Th signature cytokines IFN-γ, TGF-β, and IL-17 [69], which, together with several other earlier meta-analyses on blood cytokines, jointly imply imbalanced Th polarization in FEP patients [69–71]. Furthermore, studies have linked diminished Treg cells with negative symptoms and cognitive impairments in treatment-resistant schizophrenia [72], and elevated levels of Th-17 cells with psychopathological symptoms in drug-naïve FES patients [73].

Contrasting T-cells, no clear difference in peripheral B-cell count has been found in schizophrenia so far [74]. Closely relevant to T- and B-cell functions in autoimmunity however, autoantibodies against neuronal or non-neuronal antigens such as N-methyl-D-aspartate receptor (NMDAR) and glutamic acid decarboxylase (GAD) are reported in the blood or cerebrospinal fluid of some schizophrenia patients by a few meta-analyses [75, 76]. Patients seropositive for NMDAR autoantibodies may experience schizoaffective symptoms, memory impairment, and catatonia [75, 77, 78]. We recently reported that serum NMDAR antibody levels were associated with psychotic symptoms and cognitive impairment in FES patients [79] and also with white matter deficits in treatment-resistant schizophrenia [80].

Besides T- and B-lymphocytes, natural killer (NK) cells were also attended with interesting findings. A previous meta-analysis found increase in the percentage of NK in acutely relapsed inpatients of schizophrenia, which dropped after antipsychotic medication [67]. However, bioinformatic investigations quantifying blood cell proportions based on transcriptomic datasets from schizophrenia revealed reduced number of peripheral NK cells in schizophrenia [81] and FES patients [82]. Furthermore, another study observed an increased expression of HLA-DR in NK cells of FEP patients compared to HCs and yet a suppressed capacity of patients-derived NK cells to mount cytotoxic responses, thereby implying dampened NK activation in schizophrenia [83].

In summary, different findings suggest various types of imbalanced lymphoid populations in schizophrenia [74, 84–86], albeit the conclusions were mostly drawn from the massive measurements of Th cytokines and very few cellular functional assays in vitro and in vivo have proved solidly whether these lymphocytes are overactivated or inactivated relative to each other so far, with in fact available yet limited evidence tending to suggest impaired functions in various T-cell types, e.g., deficiency in IL-2 production and intracellular signaling in Th cells, decreased Treg proliferation [68, 86, 87] and NK cells [83] in schizophrenia.

## Potential mechanisms of immunophenotypes in psychosis: how to accommodate discrepancies?

Given the above-summarized knowledge based on studies of leukocytes both circulating in the blood and infiltrated into the brain in psychiatric disorders, immunopsychiatric researchers have kept facing the dilemmas on answering questions regarding whether immune cells are more activated or vice versa and whether they are the cause or result or by-product of psychosis in psychotic patients. This section aims to explore such key questions and search for some answers. We would like to tentatively offer some provocative ideas using psychosis or specifically FEP/FES as an example and will mainly discuss the causality, specificity, and beneficence issues of immunophenotypes of psychotic patients from the angle of heterogeneity of leukocytic population here.

### Heterogeneity of peripheral immunophenotypes in psychosis

As overviewed above, certain subclasses of blood WBC cell counts, such as neutrophils and monocytes, were found to be increased in FEP/FES patients. For peripheral biomarkers of immune cell polarization or activation, the great majority of studies are confined to cytokine signatures. Multiple meta-analyses found upregulated blood cytokines in FEP that are relevant to almost all types of macrophages or Th subtypes (e.g., M1/Th-1, M2/Th-2, Th-17, and Treg cells), pointing toward multiple cell activation models of both proinflammatory and anti-inflammatory (or in other words inflammatory and counterbalancing inflammatory) responses [69–71]. Notably, although psychosis such as schizophrenia is highly inheritable, it is also a highly heterogeneous spectrum disorder. The current literature demonstrates that a wide range of 20%~70% schizophrenia patients have altered peripheral or brain inflammatory indices [69, 88–91]. Hence, some groups have endeavored to divide schizophrenia patients into “high inflammation” versus “low inflammation” subgroups based on either peripheral blood inflammatory biomarkers such as cytokines [88, 89, 92–97] or cortical neuroinflammation-related transcripts [90, 91, 98, 99] (Table S2).

**Fig. 2:**
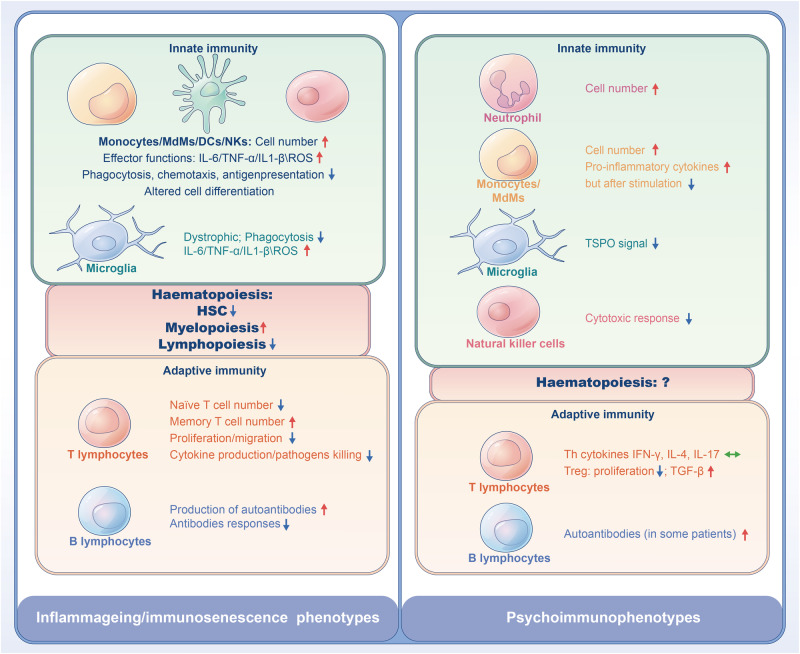
A comparison of inflamm-aging phenotypes and psychoimmunophenotypes. There are both similarities and differences between inflamm-aging and psychoimmunophenotypes. Inflamm-aging is a coin with two sides, e.g., increased myelopoiesis with concomitant decreased lymphopoiesis during aging. At cellular levels, innate immune cells are in a tonic-activated state producing more proinflammatory mediators including free radicals and proinflammatory cytokines. However, when they are stimulated, they cannot react anymore and become paralyzed/non-functional. Simultaneously, adaptive immune cells show decreased numbers of naïve T-cells with concomitant increased proportion of memory T-cells, but they are malfunctional in proliferation, migration, cytokine production, and pathogens killing. In psychosis, while the numbers of WBCs and some of their subsets including neutrophils and monocytes as well as some cytokines are increased, activation of innate cells such as monocytes and NK cells are decreased. Moreover, increase in production and release of autoantibodies as a characteristic of immunosenescence, has also been found in some patients with psychosis. DCs dendritic cells, HSC hematopoietic stem cell, IFN interferon, IL interleukin, MdMs monocyte-derived macrophages, NK natural killer, ROS reactive oxygen species, TGF transforming growth factor, TNF tumor necrosis factor, TSPO translocator protein. Green arrows represent no change, blue arrows represent downregulation, and red arrows indicate upregulation.

Some psychiatrists have proposed the remained overactive immune status of patients as a new homeostatic setpoint [68, 100], a concept resembling the term “allostatic load” that has been used to describe maladaptation to stress [101], per se a risk factor for the onset and/or relapse of psychosis [102]. Nonetheless, available cellular functional evidence provided by others and us tends to suggest dampened activations of various T-cell types [68, 86], NK cells [83], and monocyte subsets [39, 42, 43] of FEP/FES patients after being stimulated (Figs. 1 & 2). Such discrepancies imply that signs of immune activation and suppression can coexist in psychotic patients, or different blood immunophenotypes may exist in different subgroups of patients.

Also, certain autoantibodies occur in 3%~9% of schizophrenia patients and are associated with psychotic symptoms as well as cognitive dysfunction [75–79], which suggests that autoantibodies-associated autoimmune psychosis might be an important subtype. Nevertheless, a recent large study analyzing 49 anti-brain autoantibodies in over 7000 subjects showed that autoantibodies were detectable in ~17% of schizophrenia patients but also in ~15% of healthy controls, and neither seroprevalence nor immunoglobulin class or titer alone predicted disease [103]. Therefore, the dilemma that an autoantibody alone does not establish pathogenicity and is insufficient to establish an immune subtype of psychosis needs to be addressed [75, 103, 104].

### Mismatches of peripheral and central immunophenotypes in psychosis: mononuclear phagocytic cells

Likewise, the field has generated mixed scenarios regarding activation of leukocytes inside [53, 56] and outside the brain [68, 86] (Figs. 1 & 2). As summarized before, multiple meta-analyses on blood cytokines indicate rather generalized activation of the immune system in FEP, even after the resolution of acute psychosis [69–71], although some inflammatory markers may be alleviated in the stable phase of psychosis [105]. However, the prevailing inflammatory responses found in the peripheral blood are not well aligned with those in the brains of psychosis patients including FEP, as demonstrated by PET and immunohistochemistry [53, 56, 59]. In line, Weickert’s group reported that global microglial activation was not found and only CD163^+^ perivascular macrophages were increased in “high inflammation” patients [91]. They also reported that a schizophrenia subgroup with elevated inflammation displayed reduced microglial density, increased peripheral immune cells, and altered expression of neurogenesis marker gene in the subependymal zone [90]. This body of evidence doesn’t concord with the idea that cells of the mononuclear phagocytic system inside and outside the brain are synchronically activated in patients, as previously suggested by Drexhage et al. [106].

So how can such discrepancies and mismatches be explained? The decoupling phenomenon of enhanced peripheral inflammatory status without overt neuroinflammatory manifestations may have two-fold explanations. On the one hand, microglial inflammatory properties are known to be reigned and buffered by other more abundant brain cells such as astrocytes, which maybe are less vigilant and slower responders, but importantly have more capable faculty to modulate pathological stimuli compared to microglia. Astrocytic functions may still be intact in early stage of psychosis so that they behave as a strong interface between the periphery and the brain, coordinating with regulatory immune cells such as Tregs to prevent microglial overactivation [86], especially when the neurovascular unit of the blood-brain barrier is not damaged by circulatory inflammatory factors yet, as known to occur in psychosis [91, 107]. On the other hand, one may argue that peripheral changes of myeloid or lymphoid cells occurred in some FEP/FES patients, at least part of them, are merely collateral effects of brain dysfunctions in psychosis, which do not directly contribute to the pathogenesis of the disease. However, such notion is counteracted by the facts that autoimmunity contributes to psychosis in some patients [75, 77] and anti-inflammatory drugs show a favorable supplementary treatment effect in psychotic disorders, particularly for psychosis and cognitive deficits in schizophrenia [108].

Nonetheless, can other explanations exist? Psychiatric disorders are well-acknowledged to have developmental origins. Notably, emerging evidence suggests that even neurodegenerative diseases, such as Alzheimer’s disease (AD), might be largely derived from disturbed developmental programming in early life, e.g., the capacity of the brain to effectively buffer and cope with changes associated with normal aging processes and pathological damage could be developmentally registered and programmed [109]. Hence, the developmental mechanism may be applicable to the possible inflamm-aging or immunosenescence in psychiatric disorders as well. In the following sections, we will elaborate on immune system-involved developmental mechanisms that we postulate to underlie the pathophysiological processes in psychosis.

### Developmental root of psychosis: the role of glia

Abnormal brain development has been mainly attributed to psychosis, involving temporal-spatial changes in cytoarchitecture of both neurons and glia during brain development [110]. This is substantiated by evidence on genetic alterations in schizophrenia, which lead to deviation from normal developmental trajectories at early developmental stage [111], especially those involved in development of the dorsolateral prefrontal cortex [112]. Epigenetic changes during development also play a role in the aetio-pathogenesis of psychiatric disorders [113]. Remarkably, the importance of glial cells in neuronal development and establishment of local neural networks has been increasingly recognized [114]. For example, astroglial differentiation was shown to be intrinsically impaired in schizophrenia [115]. Early-life environmental risk factors, including childhood adversity and cannabis use, caused epigenetic dysregulation of biological pathways involved in inflammation and immune response in psychiatric disorders [113]. Furthermore, premature activation of microglia during fetal development hinders maturation of oligodendrocytes and astrocytes, consequently leading to compromised white matter integrity and dysfunctions in both excitatory and inhibitory neurotransmissions, typical characteristics of brain maldevelopment in schizophrenia [116]. Interestingly, we found that human embryonic microglial signature genes were more downregulated in the blood of FES patients, compared to adult microglial signature genes (submitted work).

As already mentioned, neurodegenerative diseases may be considered as late-onset neurodevelopmental disorders [109]. Abnormal development may include immune components in the degenerative brain as well. Consistently, microglia are known to exist in heterogeneous states, especially during development [117, 118], aging, and neurodegenerative diseases [119]. Interestingly, a recent study [120] found that acquisition of specific, cancer-associated driver mutations in hematopoietic stem cells from healthy aging people, which contributes to clonal hematopoiesis in inflamm-aging (see section 5.4), was protective for AD, possibly due to the effect on microglial survival or proliferation. Additionally, evidence in animal models of both familial AD and common-sporadic-type of AD demonstrated that insufficient astrocytic and microglial supports may serve as an early neurodevelopmental origin for AD pathology that manifests later in life [121, 122].

Then, could developmental mechanisms be involved in hastened senescence processes of psychosis and could aging genes exert early effects on brain structure and cognitive function in psychosis? Corroboratively, a longitudinal epigenetic study of a population at ultra-high risk for psychosis found a 2.8-fold acceleration of the epigenetic clock in converters to psychosis compared to nonconverters, due to changes in genes associated with schizophrenia and neurodevelopmental disorders [123]. Similarly, neuroimaging revealed that brain senescent acceleration occurred at early age and inflicted regions known to particularly pertain inflammation-sensitive features during development in schizophrenia patients [124, 125]. Indeed, dysregulated synaptic remodeling by microglia (and astrocytes) during maturation of neurons in adolescence contributed to cortical volume loss in schizophrenia [126]. Therefore, although still limited in knowledge, it is likely that psychosis could also have developmental root that involves aging of glial and immune cells, which warrants further investigation.

### Inflamm-aging and its developmental predisposition

Inflammation is the central pillar orchestrating the main biological pathways of aging. Senescence of the immune system, also termed ‘inflamm-aging’ (Fig. 2), is mainly characterized by chronic activation of the innate immune system, as reflected by increased myelopoiesis and low-grade sterile inflammation in the blood and localized tissues; simultaneously, inflamm-aging is also associated with decreased lymphopoiesis and immune deficiency of the adaptive immune system, resulting in aberrant response to antigens and pathogens [3–5]. As such, it turns out that chronic inflammatory condition can generate counteracting immunosuppressive state and immune tolerance, which, although necessary for the resolution of acute inflammatory conditions, is detrimental for the organism if persistent, e.g., causing immunosenescence and impaired immune memories, and inducing harmful bystander effects (i.e., spreading of senescence into neighboring cells), such as mitochondrial dysfunction and oxidative stress, in host nonlymphoid tissues/organs, especially the brain [127, 128].

Interestingly, during inflamm-aging, not only lymphoid cells contain immunosuppressive phenotypes, but functional deficiencies also appear in myeloid cells despite increased myelopoiesis, e.g., monocytes/macrophages, DCs, and NK cells [4]. For instance, inflamm-aging dysregulates monocyte subsets and their functions by increasing nonclassical monocytes yet reducing their surface expression of CX3CR1 and HLA-DR [129] and phagocytic activity [130] in human subjects. In multiple sclerosis, monocytes isolated from patients showed deficits in phagocytosis similarly as monocytes isolated from aged individuals in vitro, suggesting that monocytes in this autoimmune disease may have a prematurely aged phenotype [131]. Macrophages also age, shown as cell cycle arrest, altered differentiation, and functional impairments including chemotaxis, antigen presentation, and phagocytosis [132]. As tissue-resident macrophages, microglia are normally long-living in the young healthy brain; however, in the aged brain, microglia undergo cellular senescence similarly as macrophages and progressively show primed morphology, e.g., bigger cell bodies with short and thick cell processes, which is further exacerbated in pathological aging such as AD [133].

Evidence on the developmental root of inflamm-aging is emerging and a “stem cell hypothesis” has been proposed, which suggests that exposure to adversity during a critical developmental time window may underlie the pathogenesis of inflamm-aging [134, 135]. Supportively, senescent cells exist throughout embryonic tissues and contribute to normal development including the hematopoietic and nervous systems [136]. Genetic studies have also detected high heritability for many immune cell traits and blood cytokines/chemokines (up to 96%) in healthy individuals [137]. In addition, recombination events during the process of T-cell receptor (TCR) rearrangement and T-cell maturation within the thymus generate circular DNA fragments referred to as T-cell receptor excision circles (TRECs), which can serve as reliable indicators of thymic functionality and quantity of newly emigrating T-cells [138]. A study showed that significant reductions in circulating TRECs levels during adulthood were associated with childhood physical and emotional abuses, as well as epigenetic age acceleration [139].

Furthermore, epigenetic modifications on hematopoietic stem cells due to early-life adversities may also exert long-term effect and accelerate senescence of various immune cell types in adulthood [134]. Besides, innate immune cells, such as monocytes, macrophages, NK cells, and microglia, have demonstrated ability of forming long-term functional memory, also termed as “trained immunity”, imprinted by epigenetic reprogramming. Notably, changes in trained innate immune memory align with alterations in innate immunity observed during aging process. Thus, repeated exposure to infections or psychological stress in early life may accelerate immune “burnout” and generate a paralyzed state when exceeding a certain threshold, which is similar to the original description of inflamm-aging [2]. Hence, both genetic architecture and epigenetic modifications of it are essential in establishing specific cell identity during immune system development and may also be involved in inflamm-aging processes during adulthood and aging [63, 140].

### Can psychosis be a premature inflamm-aging disorder?

As discussed above, immunophenotypes in psychosis, either WBCs in the peripheral blood or microglia in the brain, may resemble some characteristics of inflamm-aging (Table 1 & Table S1, Fig. 2). To our surprise, however, no study has tested such hypothesis or used it to explain the immunophenotypes of psychosis in the literature so far, making it at its infantile and provocative stage currently. Aging of the immune system was only suggested for bipolar disorder earlier, albeit also with scant supporting evidence so far, such as increased low-grade proinflammatory state, increased CD8^+^CD28^-^ T-cells but decreased Tregs (reviewed in [141]).

Interestingly, recent animal studies have provided a glimpse of immune mechanisms for cognitive senescence. One study found that a group of mice genetically predisposed for accelerated senescence, generated via repetitive inbred strain breeding, exhibited an early onset of decline in the peripheral immunity, followed by brain atrophy and accompanied impairment in learning and memory when the mice reach reproductive age [142]. Another study showed that T-cells with dysfunctional mitochondria owing to mitochondrial transcription factor A (TFAM) deficiency acted as accelerators of senescence, instigating multiple aging-related features including cognitive alteration and premature death [143].

Although currently there is a lack of direct evidence revealing overlapping determinants between inflamm-aging and psychotic disorders in humans, earlier studies have indicated the genetic contribution of the *HLA* loci, the best-characterized loci attributable to psychosis [28, 29, 144, 145], to inflamm-aging as well [146–148]. Also, abnormal TCR repertoires have been identified in schizophrenia patients, at least in subtypes of them [88, 149], whereas reduction of the TCR repertoires is one of the hallmarks of T-cell aging [150]. In addition, decreased expression of CD28 on T-cells is a key indicator of T-cell aging, eventually resulting in immune incompetence [151], whereas a CD28 SNP rs3116496 may be associated with schizophrenia risk, especially in deficit patients [152]. Besides, as mentioned earlier, schizophrenia patients have shorter leukocyte telomere length, which is more pronounced in patients with greater disease severity and longer illness duration, suggesting a pathological accelerated aging profile present in schizophrenia [153–157]. Moreover, a recent multi-omics study of human plasma demonstrated molecular features of disturbed inflammation and more rapid aging in schizophrenia over lifetime. Especially, high levels of multiple cardiovascular disease biomarkers were found in patients under 40 [158].

So, could immune-developmental root also exist in psychiatric disorders, especially for those patients who do not show an enhanced peripheral immune status or even manifest a reduced one? Could their immune systems resemble a premature immune “burnout”, e.g., inflamm-aging or immunosenescence?

Agreeable to such speculations, some genetic and epigenetic findings suggest that immunobiological changes in psychosis may recapitulate inflamm-aging that initiates during early development or in later life. For instances, somatic mosaic mutations related to clonal hematopoiesis were interestingly found to be associated with all-cause mortality in schizophrenia [159]. The puberty onset of thymic involution coincided with that of schizophrenia [160]. Additionally, patients with a 22q11.2 deletion syndrome (or DiGeorge syndrome), which is manifested with T-cell deficits and bias to Th-17 due to thymic hypoplasia, are known to be ultra susceptible for schizophrenia and other psychiatric disorders [161, 162]. Furthermore, trained innate memory has been considered to contribute to pathogenesis of psychiatric disorders [63, 134] and a significant acceleration in epigenetic aging has been found in schizophrenia patients [163, 164]. Concordantly, as mentioned before, recent EWAS evidence has highlighted aberrant DNA methylations in T- and B-cell developments in schizophrenia patients [32, 33]. Intriguingly, association of decelerated epigenetic aging clock with anti-tumor NK and CD8^+^ T-cells were also reported on schizophrenia [164]. Besides, human cytomegalovirus (CMV) infection, a well-recognized trigger of brain maldevelopment in infants, may lead to psychosis through mechanisms of innate and adaptive immune activations, autoimmune cross-reactivity, and subsequent disrupted neuronal migration [165]. Importantly, CMV is also a well-known driver of immunosenescence [166]. Moreover, leukocyte telomere shortening due to CMV infection was also observed in schizophrenia [167].

Altogether, genetic predisposition along with epigenetic imprint due to early-life adversities (such as maternal or neonatal stress, hypoxia, or infection) may play an essential role in accelerating the process of immunosenescence in later life, hence affecting brain development and psychotic behaviors.

Noteworthily, compared to their monocytic counterparts, microglial maturation is known to be dependent on the immunosuppressive cytokine TGF-β [168] and they may be more susceptible to developmental impactors such as oxidative stress and DNA damage, due to the immunosuppressive microenvironment in which they have developed since embryonic age and constantly lived afterward [44, 45]. Furthermore, microglia in the psychotic brain may be less capable of regeneration under chronic inflammatory conditions that make them more vulnerable to be reprogrammed toward aging, as for example facilitated by infections, stress, or metabolic comorbidities.

**Fig. 3:**
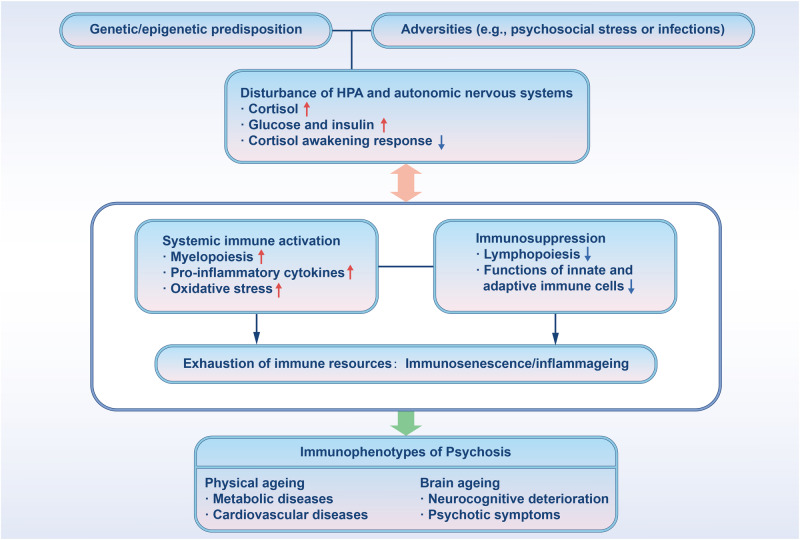
Proposed inflamm-aging-associated mechanisms underlying the pathogenesis of psychosis and comorbidities. Inflamm-aging-like changes caused by genetic and epigenetic predisposition combined with environmental risk factors may play a central role in contributing to physical aging and brain aging, which leads to psychiatric disorders and commonly associated cardio-metabolic diseases in patients. HPA: Hypothalamus-pituitary gland-adrenal gland. blue arrows represent downregulation and red arrows indicate upregulation.

Taken together, it is plausible that premature inflamm-aging may indeed occur and play a causative role in psychotic patients, which may be further facilitated or remodeled by chronic malaises. The down-tuned central immune status in psychotic patients may hence reflect a predisposed immunosenescence or immunosuppression of resident or infiltrated myeloid cells in the psychotic brain, which may have been primed/burnout in advance (Fig. 3). Noteworthily, considering inflamm-aging or immunosenescent biomarkers as additional factors may also be highly relevant for patient subgrouping research, given the fact that a wide range of schizophrenia patients have been categorized into “high versus low inflammation” subgroups (Table S2) [69, 88–99]. Besides, how much affective and non-affective psychotic disorders may overlap or differ in their inflamm-aging or immunosenescent phenotypes is still unclear at this stage. In this sense, biochemical as well as genetic and epigenetic make-up underlying inflamm-aging or immunosenescence may constitute another classification dimension for subgrouping of psychotic patients in the future differential studies.

### Inflamm-aging and psychosis: cause or result, harmful or beneficial?

As such, it is also imperative to interrogate what have been constantly inquired by psychiatric researchers: Are dysfunctional (inflamm-aged) immune cells the result of psychosis, instead of the cause? Are they harmful or beneficial, or in other words, should they be activated or inactivated?

Due to miscellaneous confounding factors such as dormant viruses, microbiome, unhealthy lifestyle, and antipsychotic medications, chronic inflammatory and metabolic diseases belong to the most prevalent comorbid conditions for psychotic patients including FEP/FES [10, 169]. Besides, these chronic immunometabolic conditions are influenced by the neuroendocrine and autonomic nervous systems [134, 170], e.g., the enhanced innate immune activity or impaired adaptive immune activity in psychosis may be caused by cortisol, which can be altered by aging [171] and is known to regulate inflamm-aging [135, 170]. Therefore, some immunophenotypes may be collateral result instead of primary cause in some patients, which may also concern our hypothesized inflamm-aging-like immunophenotypes. Nevertheless, it is still plausible that these extrinsic or intrinsic factors first drive inflamm-aging during development, which then cause the onset and exacerbation of psychosis (Fig. 3). As such, longitudinal studies on individuals at clinical ultra-high risk for psychosis have provided a distinctive perspective on the causal relationship between immune markers and psychosis. For instance, elevated peripheral blood cytokines in such individuals were associated with subsequent transition to psychosis [172, 173]. Notwithstanding, such longitudinal evidence is still very limited. Hence, addressing causal/consequential relationship between inflamm-aging and psychosis will require more high-quality large-scale longitudinal studies with careful design on patient recruitment, grouping, and multimodal immunological phenotyping.

As whether a “inflamm-aging” immunophenotype, if truly exists, is good or bad for psychotic patients, an intuitive speculation would be that chronically activated innate immune cells like M1 macrophages under inflamm-aging are harmful, whereas regulatory immune cells, such as Tregs or M2 macrophages, are regarded as neuroprotectors [86]. However, such binary stereotypic view may meet counterintuitive findings. For instance, we recently found that the plasma level of Treg signature cytokine, TGF-β1, was nearly doubled in FES patients, and its level was negatively associated with visual cortical thickness and cognitive performance [174]. TGF-β1 is one of the most commonly upregulated cytokines in FEP and known to be pleiotropic with multiple roles in inflammatory responses; although anti-inflammatory itself, together with IL-6, another most prevalently upregulated cytokine in FEP, they can promote Th-17 differentiation [175]. Another example is monocytic subsets we very recently studied in FES patients, where we found that although monocyte-related genes were overall negatively associated with cerebral cortical thickness and cognitive performance in HCs, these were surprisingly mitigated in FES patients, especially concerning nonclassical monocytes, with many genes upregulated in patients [43]. It may be so that classical monocytes were more susceptible to inflamm-aging than nonclassical monocytes, which played a compensatory role in these FES patients.

## Summary and perspectives on studying inflamm-aging in psychosis

The hint on immune components as pathological factors due to syphilis-associated psychosis and the use of immune-modulating approaches for treating psychosis occurred already more than 100 years ago [176]. In early 1990s, the macrophage-T-lymphocyte theory of schizophrenia, i.e., chronic macrophage activation with subsequent failure of activated macrophages to properly control T-lymphocyte activation, was first proposed by Smith and Maes [177]. Since then, many research results have been published confirming this theory. Although this research field rapidly evolves in the past 3 decades, clinicians and researchers are still struggling with formulating consensual theories and finding reliable biomarkers and approaches to be used in clinical diagnosis and treatment of psychiatric disorders. The still existing clinical limitations wait for immunopsychiatrists to overcome, as we briefly point out below.

As routine clinical measurements, WBCs represent feasible and useful biomarkers to study psychosis and are indeed utilized by many to unravel immune mechanisms for psychosis. However, most clinical measurements on WBCs have stopped at their total counts with few using functional cellular assays, and many studies have focused on only a certain type of immune cells using PBMCs. Hence, more sophisticated immunophenotyping approaches with advanced cutting-edge technologies are required to make ground-breaking progress in immunopsychiatry. Besides, blood inflammatory biomarkers used in most psychiatric clinics have been only conventional cytokines and chemokines, it awaits to be seen if other biochemical/genetic/epigenetic hallmarks including those of inflamm-aging can be added into the biomarker portfolio. Nevertheless, how to precisely assess inflamm-aging in psychotic disorders remains an unsettled question and would require standardized procedure borrowed from other disciplines to achieve consensual findings [127].

It is also considerable that inflamm-aging may apply to only a subset and not all psychotic patients. Indeed, subgroup propensity to immune manifestations is applicable for psychotic onset and development [29, 64], and anti-inflammatory drugs are not universally favorable in all psychotic patients [108]. Moreover, miscellaneous confounding factors, such as age, body mass index, smoking, physical inactivity, antipsychotics, and substance use, etc., may contribute to immunometabolic disturbances in patients with psychosis, which we did not address in detail due to the space limit here. Importantly, the relationship between psychosis and the immune system as well as other physiological systems are bidirectional. In this sense, careful longitudinal studies considering inclusion/exclusion criteria and patient stratification, based on such confounding factors and including those influencing inflamm-aging, would help reduce confusions and provide clearer answers to psychiatrists and patients. Additionally, it is necessary and meaningful to systematically evaluate the inflamm-aging and the accompanied immunosenescence in psychosis and high-risk individuals according to comprehensive framework for biomarkers of inflamm-aging [178, 179], such as genomic instability, telomere attrition, mitochondrial dysfunction, loss of protein homeostasis, clonal hematopoiesis, and thymic involution, etc. Moreover, future studies should more systematically report the ages of the patients and consider age subgrouping in the analyses. Besides, other therapeutic approaches such as senotherapies may be considerable to investigate in psychiatric clinical trials.

Obviously, there are still a lot for immunopsychiatrists to learn and our synthesis here may have created more questions than answers. Our hypothesis on the inflamm-aging nature of psychosis, possibly originating from developmental deviation, awaits to be tested, and the heterogeneous nature of blood leukocytes will surely make this task complicated. Nevertheless, advances in understanding the relationship of psychosis and inflamm-aging, as well as when this premature immunosenescence takes place may provide a new perspective for developing potential pharmacological therapies. Hopefully, with cross-disciplinary mindsets and current state-of-art technologies, we will be able to fill the knowledge gap in immunopsychiatry, reach to consensuses on hypothesis, and improve its clinical applications in the future.

## Supplementary information

Overview of immunophenotypes in psychosis, their association with symptoms, and their consistency to inflamm-aging phenomenon

Findings on high inflammatory subgroup of SCZ

## Data Availability

Data availability is not applicable as no datasets were generated or analyzed in this review article.
