# Supplementary material for: Immunophenotypes in psychosis: is it a premature inflamm-aging disorder?
Source: Mol Psychiatry. 2024 Mar 26;29(9):2834–48. doi: 10.1038/s41380-024-02539-z (PMC11420084; doi:10.1038/s41380-024-02539-z)
Supplement: Supplementary file 1 — Overview of immunophenotypes in psychosis, their association with symptoms, and their consistency to inflamm-aging phenomenon [file 41380_2024_2539_MOESM1_ESM.docx]

**Table S1. Overview of immunophenotypes in psychosis, their association with symptoms, and their consistency to inflamm-ageing phenomenon.**

| **Literature type** | **Study indicators** | **Results** | | | **Associations with** **clinical indices** | **Consistency to inflammageing** | **Reference (Year_PMID)** |
| --- | --- | --- | --- | --- | --- | --- | --- |
|  |  | **Significant changes** | **Increased** | **Decreased** |  |  |  |
| **Meta-analysis** (24 studies) | Total and differential WBC counts in SCZ | Total WBC, monocytes, and neutrophils （SCZ vs. HC） | √ |  | Not reported | Yes | 2020_  31850530 [1] |
|  |  | Neutrophils and monocytes (in both FEP and chronic SCZ vs. HC) | √ |  |  | Yes |  |
| **Observational study:** drug-naïve FEP patients (*n* = 129); non-first-episode SCZ patients unmedicated ≥6 weeks (*n* = 124); HC (*n* = 294) | Differential blood count, CRP, neutrophil and monocyte–macrophage activation markers, cortisol and psychotic symptoms in acute FEP and SCZ | Neutrophils, monocytes and CRP (acutely ill unmedicated FEP and SCZ patients vs. HC) | √ |  | - FEP patients with high neutrophils or monocytes had more severe positive symptoms; - CRP positively correlated with positive symptoms; - Improvement of positive symptoms after treatment correlated with declining neutrophils or CRP; - Improvement of positive symptoms after treatment correlated with rising eosinophils | Yes | 2020_ 31504969 [2] |
|  |  | Eosinophils (acutely ill unmedicated FEP and SCZ patients vs. HC) |  | √ |  | Uncertain |  |
| **Observational study:** FEP (*n* = 137); HC (*n* = 81) | Blood cells, gray matter and ventricles in FEP | Neutrophils (FEP vs. HC) | √ |  | - Neutrophil count was associated with reduced gray matter volume and increased cerebrospinal fluid volume; - Neutrophil count was also associated with the total PANSS score, including those items assessing hallucinations and avolition | Yes | 2019_ 30107610 [3] |
| **Meta-analysis** (8 studies) | NLR, MLR and PLR in non-affective psychosis | NLR and MLR (Non-affective psychosis vs. HC) | √ |  | - Not reported | Yes | 2020_  30806142 [4] |
| **Observational study:** SCZ (*n* = 9，young drug-free men with SCZ); HC (*n* = 11) | Ultrastructure of monocytes and monocyte production of IL-1β | Area of nucleolus, volume density and area of mitochondria and lysosomes, and the number of lysosomes (SCZ vs. HC) | √ |  | None  of the parameters were correlated with the points of the PANSS scale | Yes | 2017_ 28314932 [5] |
|  |  | The production of IL-1β by monocytes without stimulation (SCZ vs. HC) | √ |  |  | Yes |  |
| **Observational study:** FEP (*n* = 25); HC (*n* = 23) | Macrophage responses | Inflammatory responses of macrophages following stimulation with LPS and LPS plus IFNγ (Non-affective FEP vs. HC or affective FEP patients) |  | √ | Not reported | Yes? | 2021_ 33716670 [6] |
| **Observational study:** FES (*n* = 69, patients with normal weight, drug naïve); HC (*n* = 60) | Proportion of Th17 cells, and plasma levels of IL-17, IFN-γ and IL-6 | proportions of Th17 cells (FES vs. HC) | √ |  | - Significant positive relationships between the proportion of Th17 cells, plasma levels of IL-17, IFN-γ, IL-6 and the PANSS total score; - After 4weeks of risperidone treatment, the proportion of Th17 cells decreased significantly, and there was a significant positive relationship between the PANSS total score change rate and the change in proportion of Th17 cells | Yes | 2014_  24447943 [7] |
|  |  | plasma levels of IFN-γ and IL-6 (FES vs. HC) | √ |  |  | Yes |  |
| **Observational study:** Stable-chronic SCZ (*n* = 40); HC (*n* = 40) | Distribution of various lymphocyte subsets and Treg cells in PBMC culture；Proliferation of Treg during T cell stimulation with anti CD3/CD28 co-culture; Th1/Th2/Th17 cytokines in culture supernatants and plasma | Treg percentages in unstimulated condition (SCZ vs. HC) | √ |  | - The percentage of Treg cells was uncorrelated with clinical data； - Both CD19+ and CD20+ percentages were positively correlated with PANSS total and global scores; - The supernatant levels of IL-4 and IL-10 were negatively correlated with PANSS negative scores | Yes | 2020_ 32221694 [8] |
|  |  | Treg percentages in stimulated condition (SCZ vs. HC) |  | √ |  | No? |  |
|  |  | Activated T cell percentages in unstimulated and stimulated conditions (SCZ vs. HC) | √ |  |  | No |  |
|  |  | PBMC culture supernatant levels of IL-6, IL-17A, TNF-α and IFN-γ; and plasma levels of IL-6 and IL-17A (SCZ vs. HC) | √ |  |  | Yes |  |
| **Observational study:** FEP (*n* = 81); HC (*n* = 61) | NK cell phenotype and function | NK cell expression of HLA-DR (BP and FEP vs. HC) | √ |  | - NKG2C was positively correlated with the YMRS score in FEP; - There was an inverse correlation between NK cell IFN-γ production and PANSS scores in FEP | Yes? | 2021_  33456051 [9] |
|  |  | Expression of the activating NKG2C receptor (BP and FEP vs. HC) | √ |  |  | No? |  |
|  |  | Capacity of NK cells to mount cytotoxic responses (SCZ vs. HC) |  | √ |  | Yes |  |
| **Meta-analysis** (5 studies) | PET studies of the glial cell marker TSPO | The levels of TSPO in the frontal cortex, temporal cortex, and hippocampus, measured using total distribution volume and excluding the effect of exposure to antipsychotic treatment (FEP and SCZ vs. HC) |  | √ | Not reported | Yes? | 2018_ 29653835 [10] |
| **Meta-analysis** (13 studies) | Autoantibodies against voltage-gated potassium channel and GAD | GAD65 antibodies (Psychosis vs. HC) | GAD65 autoantibodies are more common |  | Not reported | Yes | 2017_ 28573688 [11] |
| **Observational study:** FES (*n* = 110); HC (*n* = 50) | Serum anti-NMDAR antibody | Serum anti-NMDAR antibody (FES vs. HC) | √ |  | - Positively correlated with PANSS positive, negative and total scores; - Inversely correlated with performances of verbal learning and memory, working memory, speed of processing and MCCB total scores | Yes | 2019_ 31201848 [12] |
| **Observational study:** SCZ and other psychotic disorders (*n* = 108); HC (*n* = 71)  **Meta-analysis** (18 studies) | Leukocyte telomere length after controlling for the effects of gender, age, cigarette smoking and alcohol drinking | Leukocyte telomere length (SCZ, schizoaffective disorder and psychosis not otherwise specified, < 50 years vs. HC) |  | √ | Not reported | Yes | 2018_ 30001973 [13] |
| **Observational study:** severe psychiatric disorders (*n* = 118); Non-psychiatric controls (*n* = 36) | T-lymphocyte telomere length and plasma levels of inflammatory markers | T-lymphocyte telomere length (SCZ vs. Non-psychiatric controls) |  | √ | Not reported | Yes | 2020_ 32919410 [14] |
|  |  | hsCRP (SCZ vs. Non-psychiatric controls) inversely correlated with T-lymphocytes telomere length in the whole sample | √ |  |  | Yes |  |
| **Meta-analysis** (215 studies) | Peripheral concentrations of cytokines and associated inflammatory proteins | IL-1β, IL-1RA, sIL-2R, IL-6, IL-8, IL-10, TNF-α, and CRP (in both acute and chronic schizophrenia-spectrum disorder vs. HC) | √ |  | Not reported | Yes | 2023_  36863384 [15] |
|  |  | IL-2 and IFN-γ (acute schizophrenia-spectrum disorder vs. HC) | √ |  |  | Yes |  |
|  |  | IL-4, IL-12, and IFN-γ (chronic schizophrenia-spectrum disorder vs. HC) |  | √ |  | No? |  |
| **Meta-analysis** (10 studies) | Twenty‐four peripheral cytokines | IFN‐γ, IL‐6, IL‐12 and IL‐17 (Antipsychotic-naïve FEP vs. HC) | √ |  | - IL‐1β, IL‐2, IL‐6 and TNF‐α were positively related to negative symptoms; - IL‐10 and IL-4 were negatively and positively associated with negative symptoms, respectively | Yes | 2022_ 35202480 [16] |
| **Observational study:** SCZ (*n* = 54); non-psychiatric control patients (*n* = 51) | Proteomics and metabolomics profiles in the plasma over six decades of life | Inflammatory markers (SCZ vs. Non-psychiatric controls) | √ |  | Not reported | Yes | 2022_ 34741130 [17] |
|  |  | Several known cardiovascular disease biomarkers in SCZ under 40 (SCZ vs. Non-psychiatric controls; The five most highly connected proteins in this network were C3, CST3, AHSG, CRP, and ApoE) | √ |  |  | Yes |  |

**Abbreviations:** BP, bipolar disorder; CD, cluster of differentiation; CST3, cystatin-3; FEP, first-episode psychosis; FES, first-episode schizophrenia; GAD, glutamic acid decarboxylase; HC: healthy control; HLA, human leukocyte antigen; hsCRP, hypersensitive C-reactive protein; IFN, interferon; IL, interleukin; LPS, lipopolysaccharide; MCCB, MATRICS Consensus Cognitive Battery; MLR, monocyte to lymphocyte ratio; NK, natural killers; NLR, neutrophil to lymphocyte ratio; NMDAR, N-methyl-D-aspartate receptor; PANSS, positive and negative symptoms scale; PBMCs, peripheral blood mononuclear cells; PLR, platelet to lymphocyte ratio; SCZ, schizophrenia; Th, T helper cells; TNF, tumour necrosis factor; Treg, T regulatory cells; TSPO, translocator protein; WBCs, white blood cells; YMRS, Young mania rating scale.

**References**

1. Jackson AJ, Miller BJ. Meta-analysis of total and differential white blood cell counts in schizophrenia. Acta Psychiatr Scand. 2020;142:18-26.

2. Steiner J, Frodl T, Schiltz K, Dobrowolny H, Jacobs R, Fernandes BS, et al. Innate Immune Cells and C-Reactive Protein in Acute First-Episode Psychosis and Schizophrenia: Relationship to Psychopathology and Treatment. Schizophr Bull. 2020;46:363-73.

3. Nunez C, Stephan-Otto C, Usall J, Bioque M, Lobo A, Gonzalez-Pinto A, et al. Neutrophil Count Is Associated With Reduced Gray Matter and Enlarged Ventricles in First-Episode Psychosis. Schizophr Bull. 2019;45:846-58.

4. Mazza MG, Lucchi S, Rossetti A, Clerici M. Neutrophil-lymphocyte ratio, monocyte-lymphocyte ratio and platelet-lymphocyte ratio in non-affective psychosis: A meta-analysis and systematic review. World J Biol Psychiatry. 2020;21:326-38.

5. Uranova NA, Bonartsev PD, Androsova LV, Rakhmanova VI, Kaleda VG. Impaired monocyte activation in schizophrenia: ultrastructural abnormalities and increased IL-1beta production. Eur Arch Psychiatry Clin Neurosci. 2017;267:417-26.

6. Hughes HK, Mills-Ko E, Yang H, Lesh TA, Carter CS, Ashwood P. Differential Macrophage Responses in Affective Versus Non-Affective First-Episode Psychosis Patients. Front Cell Neurosci. 2021;15:583351.

7. Ding M, Song X, Zhao J, Gao J, Li X, Yang G, et al. Activation of Th17 cells in drug naive, first episode schizophrenia. Prog Neuropsychopharmacol Biol Psychiatry. 2014;51:78-82.

8. Sahbaz C, Zibandey N, Kurtulmus A, Duran Y, Gokalp M, Kirpinar I, et al. Reduced regulatory T cells with increased proinflammatory response in patients with schizophrenia. Psychopharmacology (Berl). 2020;237:1861-71.

9. Tarantino N, Leboyer M, Bouleau A, Hamdani N, Richard JR, Boukouaci W, et al. Natural killer cells in first-episode psychosis: an innate immune signature? Mol Psychiatry. 2021;26:5297-306.

10. Plaven-Sigray P, Matheson GJ, Collste K, Ashok AH, Coughlin JM, Howes OD, et al. Positron Emission Tomography Studies of the Glial Cell Marker Translocator Protein in Patients With Psychosis: A Meta-analysis Using Individual Participant Data. Biol Psychiatry. 2018;84:433-42.

11. Grain R, Lally J, Stubbs B, Malik S, LeMince A, Nicholson TR, et al. Autoantibodies against voltage-gated potassium channel and glutamic acid decarboxylase in psychosis: A systematic review, meta-analysis, and case series. Psychiatry Clin Neurosci. 2017;71:678-89.

12. Tong J, Huang J, Luo X, Chen S, Cui Y, An H, et al. Elevated serum anti-NMDA receptor antibody levels in first-episode patients with schizophrenia. Brain Behav Immun. 2019;81:213-9.

13. Russo P, Prinzi G, Proietti S, Lamonaca P, Frustaci A, Boccia S, et al. Shorter telomere length in schizophrenia: Evidence from a real-world population and meta-analysis of most recent literature. Schizophr Res. 2018;202:37-45.

14. Squassina A, Manchia M, Pisanu C, Ardau R, Arzedi C, Bocchetta A, et al. Telomere attrition and inflammatory load in severe psychiatric disorders and in response to psychotropic medications. Neuropsychopharmacology. 2020;45:2229-38.

15. Halstead S, Siskind D, Amft M, Wagner E, Yakimov V, Shih-Jung Liu Z, et al. Alteration patterns of peripheral concentrations of cytokines and associated inflammatory proteins in acute and chronic stages of schizophrenia: a systematic review and network meta-analysis. Lancet Psychiatry. 2023;10:260-71.

16. Dunleavy C, Elsworthy RJ, Upthegrove R, Wood SJ, Aldred S. Inflammation in first-episode psychosis: The contribution of inflammatory biomarkers to the emergence of negative symptoms, a systematic review and meta-analysis. Acta Psychiatr Scand. 2022;146:6-20.

17. Campeau A, Mills RH, Stevens T, Rossitto LA, Meehan M, Dorrestein P, et al. Multi-omics of human plasma reveals molecular features of dysregulated inflammation and accelerated aging in schizophrenia. Mol Psychiatry. 2022;27:1217-25.
