# Supplementary material for: Immunophenotypes in psychosis: is it a premature inflamm-aging disorder?
Source: Mol Psychiatry. 2024 Mar 26;29(9):2834–48. doi: 10.1038/s41380-024-02539-z (PMC11420084; doi:10.1038/s41380-024-02539-z)
Supplement: Supplementary file 2 — Findings on high inflammatory subgroup of SCZ [file 41380_2024_2539_MOESM2_ESM.docx]

**Table S2. Findings on high inflammatory subgroup of SCZ**

| Cohort | Blood or brain biomarkers | Patients’ subgrouping method | Prevalence of high inflammation | Findings on high inflammation subgroup | Reference  (Year_ PMID) |
| --- | --- | --- | --- | --- | --- |
| FEP High (*n* = 27), FEP Low (*n* = 86 + 14), and HC (*n* = 3 + 70 + 44) | Twelve blood transcripts: *IL1β, IL12A, IL2RA, IFNG, IFNA21, ENTPD1, CXCR3, GATA3, CCR7, PTX3, MXD1 and MRC1/CD206* | An unsupervised machine learning model | ~21% | - A subgroup clustered with high expression of inflammatory and immune-activating genes (*IL1β*, *CCR7*, *IL12A*, and *CXCR3*) than an immune balanced subgroup; - Not related to specific symptoms; - FEP patients included in the balanced immune subgroup showed a thinning of the left supramarginal and superiorfrontal cortex | 2023_ 36604602 [1] |
| Psychosis Proband High (*n* = 29), Psychosis Proband Low (*n* = 65), HC Low (*n* = 32), | Thirteen blood biomarkers: IL1β, IL6, IL8, IL10, IL12/IL23p40, IFNγ, TNFα, TNFβ, CRP, Flt-1, VEGF, VEGFD, and C4a (IL1α, IL2, IL4, IL12p70, VEGFC, and TGFβ1 were excluded) | PCA and subsequent hierarchical clustering (PC1 loaded highly by CRP, IFNγ, IL1β, IL8, IL10, TNFα, and VEGF) | 31% | - Higher levels of 10 cytokines (except TNFβ and VEGFD) and C4a than HCs and Low group; - Lower anterior Default Mode network activity and functional connectivity than Low group; - Correlation of lower Default Mode network activity with worse cognition | 2023_37506949 [2] |
| FES High (*n* = 17), FES Low (*n* = 30), and HC (*n* = 33) | Twelve blood biomarkers: IL1β, IL6, IL8, IL10, IL12/IL23p40, IFNγ, TNFα, sFlt-1, BDNF, bFGF, PlGF, and VEGF | PCA and subsequent hierarchical clustering (PC1 loaded for IL10, IL1β, IL6, IL8, TNFα, PlGF, VEGF, bFGF, sFlt-1, and BDNF) | 36% | - Higher IL1β, IL6, IL8, and TNFα than Low group and higher levels of IL1β and IL8 than HCs; - Greater cortical thickness in the right parahippocampal, caudal anterior cingulate, and bank superior sulcus than Low group | 2022_34875344 [3] |
| Psychosis Proband High (*n* = 51), Psychosis Proband Low (*n* = 89), HC (*n* = 60), | Thirteen blood biomarkers: IL1β, IL6, IL8, IL10, IL12/IL23p40, IFNγ, TNFα, TNFβ, CRP, Flt-1, VEGF, VEGFD, and C4a (IL1α, IL2, IL4, IL12p70, VEGFC, and TGFβ1 were excluded) | PCA and subsequent hierarchical clustering (PC1 loaded highly by CRP, IFNγ, IL1β, IL8, IL10, TNFα, and VEGF) | 36% | - Higher levels of 10 cytokines (except TNFβ and VEGFD) and C4a than HCs and Low group; - Greater volumes in the bilateral hippocampus, amygdala, and putamen, as well as the left thalamus and increased thickness in several gray matter regions than Low group; - Worse visuo-spatial working memory and response inhibition | 2021_33060818 [4] |
| SCZ High (*n* = 264), SCZ Low (*n* = 79), and HC (*n* = 200 + 570) | Twenty-two blood biomarkers:  SERPINA3, GFAP, NSE, A2M, S100B, BAFF, APRIL, ICAM-1, VCAM-1, MadCAM-1, P-selectin, JAMA, IL-18BP, IL-18R1, 18RAP, HNP1-3, BD-1, BD-2, GROα/CXCL1, SDF1α/CXCL12, Eotaxin/CCL11, RANTES/CCL5 | Canonical correlation analysis and subsequent hierarchical clustering | 76% | - Cluster 1 had higher IL-18, IL-18BP and BD-2 levels, lower VCAM-1 levels, and lower cognitive scores on verbal learning and psychomotor processing speed than cluster 2; - Cluster 1 had lower IQ and years of education, and higher age, CRP, and BMI than cluster 2; - Cluster 1 had lower functioning and more severe psychosis than cluster 2 | 2022_ 36577840 [5] |
| SCZ High (*n* = 43), SCZ Low (*n* = 47), and HC (*n* = 25 + 50) | Five blood transcripts:  *IL1β*, *IL2*, *IL6*, *IL8*, and *IL-18* | Recursive two-step clustering | 48% | - Higher mRNAs of *IL1β*, *IL2*, *IL6*, *IL8*, and *IL-18*; - Higher plasma IL-8 than Low group; - The mean increase in the proportion of the schizophrenia group with elevated cytokines compared with the control group was 11.4% ± 4.6 | 2017_ 28923068 [6] |
| SCZ High (*n* = 17), SCZ Low (*n* = 26), and HC (*n* = 9 + 33) | Five blood transcripts:  *IL1β*, *IL2*, *IL6*, *IL8*, and *IL-18* | Recursive two-step clustering | 39% | - Smaller Broca’s area; - Worse verbal fluency | 2015_ 26194183 [7] |
| SCZ Hypermethylation (*n* = 25), SCZ Hypomethylation (*n* = 38), and HC (*n* = 59) | Blood whole-genome DNA methylation profiles | Non-negative matrix factorization and k-means clustering | 39.7% | - Widespread methylation level alterations among genes enriched in immune cell activity, as well as a higher proportion of neutrophils and lower proportion of lymphocytes; - higher symptom severity; - worse on cognitive measures; - greater reductions in fractional anisotropy of white matter tracts and gray matter thickening | 2021_34588622 [8] |
| SCZ High (*n* = 11), SCZ Low (*n* = 19), and Controls (*n* = 32) | Four brain transcripts: *IL6, IL6R, IL1R1* and *SERPINA3* | Pilot two-step clustering | 37% | - mRNAs of markers for phagocytic microglia were reduced; - mRNAs of markers for perivascular macrophages, pro-inflammatory macrophages, monocytes, natural killer cells and adhesion molecules were increased; - quiescent stem cell marker mRNA was reduced; - neuronal progenitor and immature neuron marker mRNAs were decreased | 2021_ 34911938 [9] |
| SCZ High (*n* = 14), SCZ Low (*n* = 23), and Controls Low (*n* = 33) | Four brain transcripts: *IL6*, *IL8*, *IL1β*, and *SERPINA3* | Recursive two-step clustering | ~40% | - lower ABCG2 and higher ICAM1, VE-cadherin, occludin and interferon-induced transmembrane protein mRNAs； - Elevated immune cell mRNAs in brain tissue, including *CD14, CD16* and *CD163*； - CD163+ perivascular macrophages existed in brain parenchyma in over 40% of "high inflammation" SCZ | 2020_ 30214039 [10] |
| SCZ High (*n* = 15), SCZ Low (*n* = 20), and Controls (*n* = 6 + 29) | Six brain transcripts:  *IL1β*, *IL1RL1*, *IL6*, *PTGS2*, *TNF*, and *SERPINA3* | Recursive two-step clustering | 43% | - Higher *IL1RL1*, *IL6*, *TNF*, and *SERPINA3* mRNAs than Low group; - Higher HSP and lower GR stress gene mRNAs than Low group | 2014_ 24569695 [11] |
| SCZ High (*n* = 14), SCZ Low (*n* = 23), and Controls (*n* = 4 + 34) | Four brain transcripts: *IL6*, *IL8*, *IL1β*, and *SERPINA3* | Recursive two-step clustering | ~40% | - Higher *IL6*, *IL8*, *IL1β*, and *SERPINA3* mRNAs as well as lower *BDNF* and *Somatostatin* mRNAs than Low group | 2013_ 22869038 [12] |

**Abbreviations:** FEP, first-episode psychosis; FES, first-episode schizophrenia; HC: healthy control; PCA, principal component analysis.

**References**

1. Enrico P, Delvecchio G, Turtulici N, Aronica R, Pigoni A, Squarcina L, et al. A machine learning approach on whole blood immunomarkers to identify an inflammation-associated psychosis onset subgroup. Mol Psychiatry. 2023;28:1190-200.

2. Lizano P, Kiely C, Mijalkov M, Meda SA, Keedy SK, Hoang D, et al. Peripheral inflammatory subgroup differences in anterior Default Mode network and multiplex functional network topology are associated with cognition in psychosis. Brain Behav Immun. 2023;114:3-15.

3. Hoang D, Xu Y, Lutz O, Bannai D, Zeng V, Bishop JR, et al. Inflammatory Subtypes in Antipsychotic-Naive First-Episode Schizophrenia are Associated with Altered Brain Morphology and Topological Organization. Brain Behav Immun. 2022;100:297-308.

4. Lizano P, Lutz O, Xu Y, Rubin LH, Paskowitz L, Lee AM, et al. Multivariate relationships between peripheral inflammatory marker subtypes and cognitive and brain structural measures in psychosis. Mol Psychiatry. 2021;26:3430-43.

5. Saether LS, Ueland T, Haatveit B, Maglanoc LA, Szabo A, Djurovic S, et al. Inflammation and cognition in severe mental illness: patterns of covariation and subgroups. Mol Psychiatry. 2023;28:1284-92.

6. Boerrigter D, Weickert TW, Lenroot R, O'Donnell M, Galletly C, Liu D, et al. Using blood cytokine measures to define high inflammatory biotype of schizophrenia and schizoaffective disorder. J Neuroinflammation. 2017;14:188.

7. Fillman SG, Weickert TW, Lenroot RK, Catts SV, Bruggemann JM, Catts VS, et al. Elevated peripheral cytokines characterize a subgroup of people with schizophrenia displaying poor verbal fluency and reduced Broca's area volume. Mol Psychiatry. 2016;21:1090-8.

8. Luo C, Pi X, Hu N, Wang X, Xiao Y, Li S, et al. Subtypes of schizophrenia identified by multi-omic measures associated with dysregulated immune function. Mol Psychiatry. 2021;26:6926-36.

9. North HF, Weissleder C, Fullerton JM, Sager R, Webster MJ, Weickert CS. A schizophrenia subgroup with elevated inflammation displays reduced microglia, increased peripheral immune cell and altered neurogenesis marker gene expression in the subependymal zone. Transl Psychiatry. 2021;11:635.

10. Cai HQ, Catts VS, Webster MJ, Galletly C, Liu D, O'Donnell M, et al. Increased macrophages and changed brain endothelial cell gene expression in the frontal cortex of people with schizophrenia displaying inflammation. Mol Psychiatry. 2020;25:761-75.

11. Fillman SG, Sinclair D, Fung SJ, Webster MJ, Shannon Weickert C. Markers of inflammation and stress distinguish subsets of individuals with schizophrenia and bipolar disorder. Transl Psychiatry. 2014;4:e365.

12. Fillman SG, Cloonan N, Catts VS, Miller LC, Wong J, McCrossin T, et al. Increased inflammatory markers identified in the dorsolateral prefrontal cortex of individuals with schizophrenia. Mol Psychiatry. 2013;18:206-14.
